# Highlighting variability in fentanyl test strip instructions using thematic content analysis

**DOI:** 10.1186/s12954-025-01252-6

**Published:** 2025-06-24

**Authors:** Cianna J. Piercey, Claire L. Pince, Hollis C. Karoly

**Affiliations:** 1 Department of Psychology, Colorado State University, 1876 Campus Delivery, Fort Collins, CO 80523-1876 USA; 2 Department of Psychiatry, School of Medicine, University of Colorado Anschutz Medical Campus, Aurora, CO USA

**Keywords:** Fentanyl test strips, Fentanyl test strip instructions, Harm reduction, Public health communication, Drug checking

## Abstract

**Background:**

Fentanyl test strips (FTS) are a harm reduction tool used by individuals seeking to avoid unintentional fentanyl exposure while consuming other illicit substances (e.g., heroin, cocaine). While evidence speaks to the efficacy and acceptability of FTS, there are currently no standardized instructions for the use of FTS as a drug checking tool, and little is known about potential variability across instructions.

**Methods:**

We sought to investigate variability in content across FTS instructions (*N* = 16) through conducting a thematic content analysis of instructions listed in the first three pages of a Google search. The search was conducted in May of 2024, with “fentanyl test strip instructions” entered as the search term. To be included in the present analysis, the information listed in the search result must have contained explicit instructions for how to use FTS and have been printed in English.

**Results:**

Thematic content analysis of FTS instructions yielded 26 codes and 4 themes. Themes included (1) Information about FTS (2) Testing Methods (3) Test Results and (4) Additional Resources. Overall, results indicated considerable variability across the 16 instructions examined, with the greatest variability observed within the testing methods theme.

**Conclusion:**

Inconsistencies in online FTS instructions, such as those identified in the current study, could lead to distrust among people who use drugs and disengagement with this drug checking practice. Standardized and accessible instructions are critical to optimizing the efficacy of FTS as a harm reduction tool and reducing accidental fentanyl exposure.

## Introduction

Over 100,000 overdose deaths were reported by the Centers for Disease Control and Prevention in 2023, and drug-related fatalities remain a leading cause of death among adults in the United States [1]. Nonpharmaceutical synthetic opioids, namely illegally manufactured fentanyl and fentanyl analogues, have been implicated in the majority of overdose deaths in recent years, often attributed to their strong potency and rapid onset of effects [2]. Further, there have been a number of deaths linked to unintentional fentanyl exposure occurring via consumption of other illicit substances (e.g., heroin, cocaine, methamphetamine) that are adulterated or contaminated with fentanyl [3].

In response to concerns related to fentanyl contamination and adulteration, fentanyl test strips (FTS) have emerged as a harm reduction tool for individuals seeking to avoid unintentional fentanyl exposure while consuming other drugs [4]. FTS are immunoassay tests that were originally developed to detect the presence of fentanyl in urine but have increasingly been used by people who use drugs to check their drug sample for the presence of fentanyl prior to consumption. Recent evidence suggests that FTS can detect most fentanyl analogues, but accuracy appears to be highly dependent on the dilution with which test samples are prepared [4]. Specifically, fentanyl test strip instructions direct people who use drugs to dilute their drug sample in water (either a portion of the sample or the full sample), allowing them to insert the immunoassay test into the solution and obtain a positive or negative test result. Notably, dilution procedures also vary across the substance being tested with FTS (e.g., some instructions recommend a greater dilution for 3–4 methylenedioxymethamphetamine [MDMA] and methamphetamine than for other substances) [5].

Despite the importance of dilution procedures related to FTS use, there are currently no standardized instructions for use of FTS as a drug checking tool and there is little regulatory oversight of FTS for harm reduction purposes. Additionally, little is known about the variability in FTS instructions distributed across organizations or the extent to which these instructions are accessible to people who use drugs. Understanding variability in FTS instructions is a critical first step in developing standardized and accessible instructions for individuals engaged with this increasingly common harm reduction practice. Thus, we sought to understand variability in FTS instructions through conducting a thematic content analysis of FTS instructions available online.

## Methods

### Search strategy

To obtain FTS instructions for analysis, we conducted a Google search with “fentanyl test strip instructions” entered as the search term in May 2024. We then extracted FTS instructions from the first three pages of results listed on Google (producing a total of 16 sets of instructions). The search took place in Fort Collins, Colorado, thus results were limited to North America, as Google considers user location when ranking search results. This search strategy was employed to ensure that instructions were representative of the information that is most readily available to US-based consumers online, particularly given evidence suggesting that most people do not read past the first page of online search results [6]. To be included in the present analysis, the information listed in the search result must have contained explicit instructions for how to use FTS and have been printed in English. Most instructions (*N* = 16) were from U.S. governmental organizations at the federal (*N* = 1), state (*N* = 11), county (*N* = 1), and city level (*N* = 1), with the remaining instructions being from a Canadian governmental agency (*N* = 1) and a U.S.-based harm reduction organization (*N* = 1) respectively.

### Analysis of FTS instructions

FTS Instructions were coded in the qualitative analysis software MAXQDA 24.3 [7], following recommendations from Dengah et al. [8]. Specifically, PDF files of the extracted FTS instructions were imported into MAXQDA and a set of codes were created by the first and second author using an open-coding approach. An open-coding approach was used to generate codes given the lack of empirical evidence evaluating FTS instructions to-date [9]. Each code was assigned an agreed upon description or “memo” to aid in the coding process and maximize consistency across coders [8]. Themes were then generated following recommendations from Ryan and Bernard [10], with a specific focus on repetitions in the data and similarities and differences across instructions. The first and second author then engaged in independent focused coding of each set of FTS instructions. After independent coding, the first and second author met to resolve discrepancies in codes until 100% agreement was reached between coders.

## Results

**Fig. 1:**
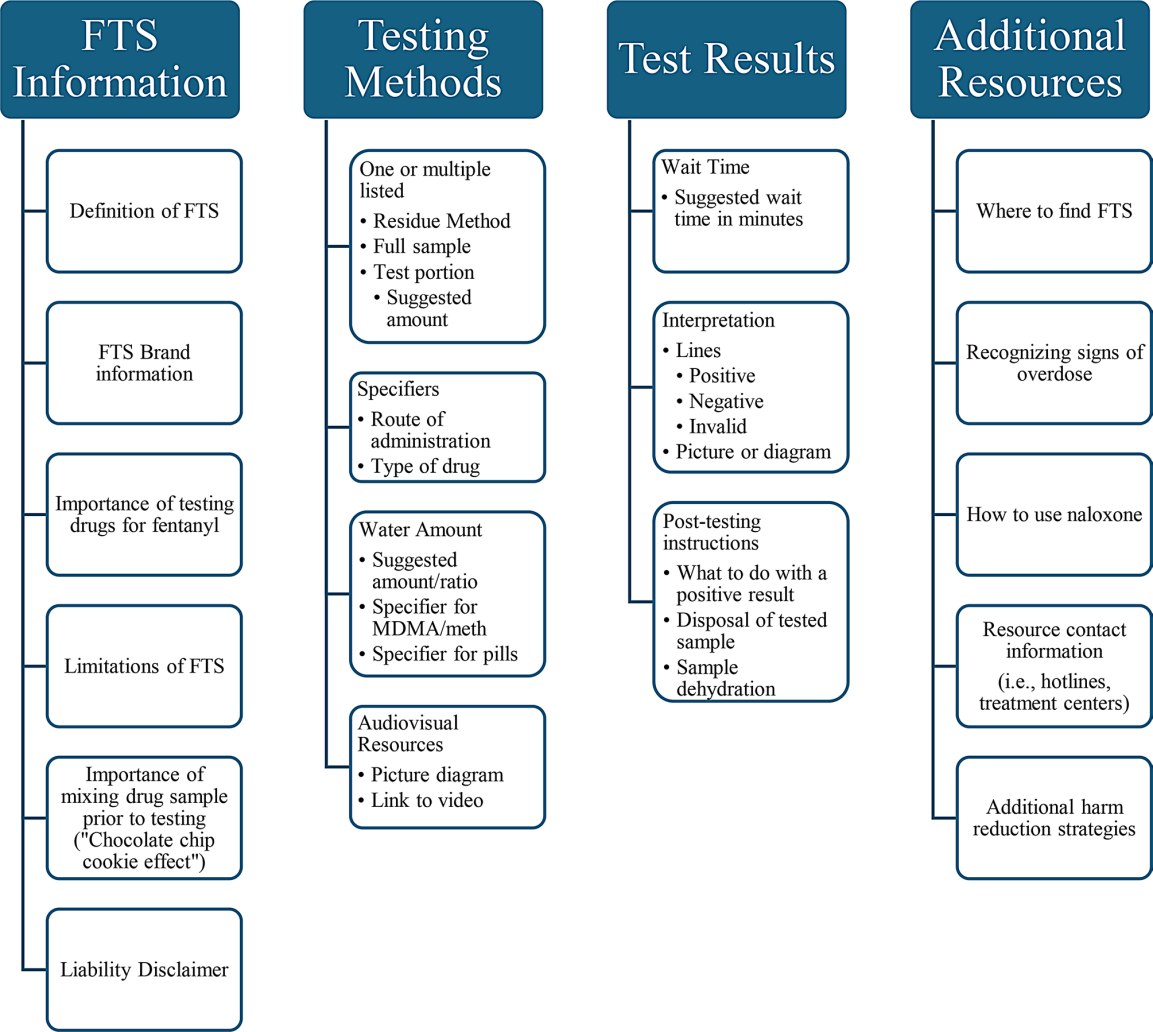
Themes generated during analysis of fentanyl test strip instructions and respective codes

Open coding of FTS instructions yielded 26 codes (Table 1) and 4 themes (Fig. 1). Generated themes were (1) Information about FTS (2) Testing Methods (3) Test Results and (4) Additional Resources.

**Table 1:** Codes generated during analysis of fentanyl test strip instructions

	Codes
1	definition of FTS
2	importance of using FTS
3	mention of a specific FTS brand
4	liability disclaimer
5	limitations of FTS
6	mention of the “chocolate chip cookie effect” (i.e., importance of mixing drug sample to evenly distribute fentanyl before testing)
7	discussion of only one testing method
8	discussion of multiple testing methods
9	specification between routes of administration
10	specification between different substances or drug classes
11	“dissolve full sample” method (i.e., entire drug sample is tested)
12	“test a portion” method (i.e., only a small part of the drug sample is tested)
13	“residue” method (i.e., only drug residue from a baggie or container is tested)
14	time to insert test strip in solution
15	amount of water to use
16	additional audio/visual information
17	length of time to wait for test results
18	information about interpreting test results
19	instructions to discard testing solution
20	instructions to dry out drug sample after testing
21	guidance for positive test results
22	where to obtain FTS
23	how to use naloxone
24	how to recognize signs of an overdose
25	additional harm reduction strategies
26	links to additional resources

### Theme 1: information about FTS

Theme 1, Information about FTS, encompassed codes 1–6. Half of the instructions analyzed (*N* = 8) provided a definition of FTS prior to outlining specific steps for using FTS to test one’s drugs for fentanyl. Additionally, over two-thirds of the instructions (*N* = 11) noted the importance of using FTS, often detailing information surrounding the presence of fentanyl in the illicit drug supply and the potency of fentanyl in comparison to other drugs. However, information about limitations of FTS (*N* = 9) was somewhat less prevalent, with two sets of instructions providing a liability disclaimer. Just over a third of instructions (*N* = 6) mentioned a specific FTS brand. Finally, some instructions (*N* = 7) provided information about how fentanyl might be distributed in an adulterated drug sample. Specifically, fentanyl may not be distributed uniformly in a contaminated or adulterated drug sample, a phenomenon that has colloquially become known as the “chocolate chip cookie effect”.

### Theme 2: testing methods

Theme 2, Testing Methods, encompassed codes 7–16. Procedures outlined for the use of FTS varied across instructions, with some instructions detailing multiple testing methods (*N* = 7) and others providing an overview of just one testing method (*N* = 9). We identified three methods specified across FTS instructions, which we coded as the “residue method” (i.e., using FTS on only the drug residue left in a bag), the “test a portion method” (i.e., using FTS on only a small amount of one’s drug sample) and the “dissolve full sample method” (i.e., using FTS on one’s entire drug sample prior to use). The “test a portion method” was cited most frequently (*N* = 12), followed by the “residue method” (*N* = 9). However, the “dissolve full sample method” was outlined in just 3 sets of instructions.

Regarding the “test a portion” method, the instructions varied substantially in the amount of drug sample recommended for testing, with specifications ranging from 10 mg, 50 mg, “a few grains”, to instances in which no amount was specified. Half of instructions (*N* = 8) provided testing information specific to intended route of administration (e.g., injection, insufflation, oral administration). For example, one set of instructions recommended use of the “test a portion” method for drugs that will be injected, the “residue method” for drugs that will be insufflated, and the “dissolve full sample” method for drugs that will be consumed orally (e.g., pills). Additionally, just over half of instructions (*N* = 9) differentiated testing procedures based on the substance being tested. In particular, due to the documented potential for false positives when testing some stimulants with certain brands of FTS [5], instructions that differentiated dilution procedures by substance often recommended to use a greater amount of water for MDMA and methamphetamine (and in one case Adderall) or to use less product (i.e., increasing the ratio of water to drug sample), but inconsistencies with regard to dilution ratio were observed (e.g., some instructions specified to use a teaspoon of water per 10 mg of drug sample, while others stated to use “up to half a cup” of water). Further, one set of instructions recommended to dilute cocaine in a “very small amount of water,” but did not specify an amount. Other substance specific recommendations included finely crushing pills and soaking blotter LSD in water prior to testing. Most (*N* = 14), but not all, instructions provided information about the amount of water to be used for testing. However, recommendations for the amount of water to use varied between instructions, even when accounting for instructions that differentiated dilution procedures by substance. Most instructions specified an amount of time to insert the test strip (*N* = 15) in the testing solution, with all instructions consistently recommending 15 s. Roughly two thirds of instructions (*N* = 10) incorporated additional audio or visual aids, such as links to videos or picture diagrams.

### Theme 3: test results

Theme 3, Test results, encompassed codes 17–21. All 16 sets of instructions specified a length of time to wait for test results after performing the test, however, wait times again varied across instructions. For example, some instructions called for waiting as little as 2 min and others as long as 10 min, whereas some instructions simply called for waiting “until results appear.” All 16 sets of instructions also provided information about how to interpret a positive (one line) or negative test (two lines) result, while 11 instructions provided information about invalid test results and 10 instructions provided a picture diagram for interpreting results. Four instructions noted the possibility of a faint second line when interpreting test results and one set of instructions (DanceSafe brand) highlighted the possibility of a faint second line emerging and then subsequently fading away if results were read prior to the directed wait time, both issues which could impact interpretation of test results. Additionally, less than half of the instructions (*N* = 7) provided guidance related to positive test results. Among instructions that included this information, guidance typically included strategies for reducing risk, such as “start with a smaller amount than usual” and “have naloxone ready.” Finally, two instruction sets provided information about how to discard one’s drug sample following testing, while one set of instructions provided information on how to dry out one’s drug sample for later use.

### Theme 4: additional resources

Theme 4, Additional Resources, encompassed codes 22–26. Several instructions provided additional resources after outlining testing procedures, such as where to obtain FTS (*N* = 4), how to use naloxone (*N* = 1), and how to recognize the signs of an overdose (*N* = 1). Most instructions (*N* = 13) also included additional harm reductions strategies to be used in conjunction with drug checking. Lastly, 11 sets of instructions included links or QR codes to additional harm reduction resources, contact information for crisis and spotting hotlines, and/or information about seeking treatment for substance use and addiction.

## Discussion

We sought to investigate variability in content across 16 FTS instruction documents through conducting a thematic content analysis of instructions from websites featured in the first three pages of a Google search conducted in May of 2024. Results highlighted several points of convergence and divergence across organizations and four themes were generated from the data.

Findings underscore a potential need for FTS instructions to educate consumers more consistently regarding the utility and importance of using FTS, associated limitations, and brand information. Half of the instruction sets provided a general overview of FTS (*N* = 8) and roughly two thirds emphasized why using FTS could be an important harm reduction tool (*N* = 11). Several instructions emphasized the potency of fentanyl, however few provided statistics about overdose mortality rates (*N* = 2). While some research suggests that sharing quantitative information can promote trust in and adherence to public health recommendations [11], individuals searching for FTS instructions online may already be aware of risks associated with fentanyl use—though, including this information could be particularly important for engaging individuals who are ambivalent or hesitant about using FTS. Perhaps of more notable concern are inconsistencies observed in the reporting of FTS limitations (e.g., lot-to-lot variability in FTS, potential for false positives and negatives) [12], as exclusion of this information could result in overconfidence in test results or potentially even disengagement with other harm reduction strategies [13–18]. Additionally, only a third of instruction sets indicated a specific FTS brand. Critically, brand-to-brand variability in performance and vulnerability to interferences could lead to testing inaccuracies when this information is not included [19].

The most notable variability in FTS instructions was found within the second theme “Testing Methods.” Instructions ranged drastically regarding the types of methods reported, with some documents including only one testing method (*N* = 9) and others including much more detail across multiple methods. Despite a recent increase in US overdose deaths involving both stimulants and fentanyl [20], only about half of analyzed documents differentiated dilution recommendations based on the substance being tested (*N* = 9) and intended route of administration (*N* = 8). Additionally, substantial differences in the recommended amount of water to use for dilution were observed. Critically, these variations could lead to confusion among individuals attempting to use FTS as a harm reduction tool, which has the potential to result in serious harm. For example, inaccuracies in dilution procedures could potentially cause highly potent fentanyl analogues (e.g., carfentanil) to go undetected (i.e., false negatives), with evidence suggesting that FTS results are highly concentration dependent [4]. Further, research suggests that for stimulants such as methamphetamine and MDMA, too high a concentration of these substances could lead to false positives [5], which has the potential to reduce trust in FTS as a harm reduction tool. There was a notably wide range of suggested water amounts (1 teaspoon to ½ cup of water) in the instruction sets for samples containing MDMA or methamphetamine. Additionally, concern over wasting one’s drug sample due to testing has been identified as a barrier to FTS use in qualitative literature [21–24]. As two instruction sets advised disposing of the sample solution after testing and one provided instructions for drying out one’s drug sample for later use, further research is needed to clarify the safety and utility of providing instructions for sample dehydration after using FTS to reduce barriers to use [25].

With regard to theme 3 (“Test Results”), wait times varied between 2 and 10 min, and while all instructions included guidelines for interpreting positive and negative test results, some instructions did not include guidelines for recognizing invalid results (*N* = 5), which may occur for a variety of reasons (e.g., if the strip is wetted above the solid test line), or visual aids to assist in interpretation of results (*N* = 6). Research on effective communication of health information suggests that the use of images along with plain language written text can improve attention, recall, comprehension, and overall readability of materials [26–28], thus the use of visual aids for interpreting FTS results may serve to improve comprehension as well as increase accessibility across literacy levels. Furthermore, standardizing wait times and interpretation guidelines is critical for enhancing consumer trust in FTS and even more importantly, justifiable confidence in the accuracy of test results. There is evidence to suggest that typically, test results should appear within 5 min [19], however variability in time for the test strip to show a positive result is not uncommon, which may be related to the concentration of the sample tested [21]. This call to action is supported by recent qualitative findings, which have revealed confusion in interpreting test results as a perceived barrier to using FTS [21, 25].

While many FTS instructions included additional resources (theme 4), inclusion of additional strategies varied in their breadth and specificity. While most instructions (*N* = 13) presented harm reduction strategies such as never using alone, carrying naloxone, and taking small amounts to start, only half explicitly referred to guidance around what to do if a sample tests positive for fentanyl (*N* = 7). Although FTS may help people who use drugs make more informed decisions about their substance use, FTS cannot mitigate all substance-related risks. Thus, inclusion of these supplemental strategies may serve as an important reminder to engage in a comprehensive approach to safer substance use, regardless of whether a sample tests positive for fentanyl. More broadly, inclusion of supplementary harm reduction strategies alongside fentanyl test strip instructions may be a pragmatic way of distributing this information to people who use drugs. However, it may also be important to consider that inclusion of too much supplementary material could lead to cognitive overload [29, 30], potentially detracting from the intended purpose of including such information.

It is worth noting that few instructions included manufacturer/brand information (*N* = 6), which created challenges in distinguishing between variability resultant from inconsistent instructional quality and variability attributable to differences across FTS brands. BTNX has historically been the most common brand of FTS available, however the market for FTS has expanded significantly over the past several years in response to the overdose crisis [31]. While this has made drug-checking supplies more widely accessible, there have also been concerns associated with expanding manufacturing without adequate regulation and enforcement mechanisms in place [31]. For example, recent studies have revealed significant lot-to-lot variability as well as variability between FTS brands in sensitivity to fentanyl, analogs, and known interferences [12, 19, 31–33].

Variability stemming from brand differences may be expected for certain instructional content due to differences in FTS manufacturing processes [31, 34]. With regard to instructional content identified in the present analysis, brand of FTS may be most relevant to themes two (testing methods) and three (test results), and specifically codes 14 (time to insert the test strip into the solution), 15 (amount of water to use), 17 (length of time to wait for test results), and 18 (information about interpreting test results). However, brand of FTS is likely less relevant to variability uncovered within themes one (information about FTS) and four (additional resources), which includes information such as the importance of using FTS (code 2) and additional harm reduction strategies (code 25). Importantly, limitations of FTS (code 5) may be both general (e.g., FTS cannot determine the concentration of fentanyl or fentanyl analogues within a drug sample) and brand-specific (e.g., reduced sensitivity of a particular brand to certain analogs) [32].

Although several instructions acknowledge limitations of FTS and in some cases even present a liability disclaimer, FTS instructions can only be as reliable as the strips themselves. This points to an overarching need for greater regulation in FTS manufacturing and standardization of instructional content across brands to ensure consumer safety [35]. In line with this need, the harm reduction organization DanceSafe recently launched a new brand of FTS manufactured by WHPM, with the express goal of improving FTS sensitivity, specificity, and quality control [36]. However, as FTS continue to evolve as a harm reduction tool, recent works suggests it may be beneficial to check drugs with multiple FTS brands to circumvent “blind spots” in detection [32], a practice which was not reflected in any of the instructional materials examined, but that may warrant further consideration in terms of practical application.

### Limitations and future directions

Findings are limited in part by the search strategy employed, as Google considers a variety of factors when ranking search results, including user location and relevance to search terms. Additionally, results are limited to information available through an online search and may not be representative of FTS instructions acquired in person from community organizations (e.g., needle and syringe programs). Future studies may consider building upon this work by using different search engines or combinations of key words to develop a comprehensive understanding of the quality and accessibility of FTS instructions online, as well as collaborating with community organizations to obtain FTS instructions available through offline modalities. Future work may also benefit from analyzing instructions available directly from FTS manufacturers or employing stratified sampling methods to obtain FTS instructions from diverse geographic regions across the US, which could then potentially be used to draw comparisons in the content of FTS instructions across varying sociocultural contexts and drug markets. Our inability to stratify results by manufacturer was another limitation of the present analysis. Future work would benefit from intentional sampling of instructions that include brand information to better discriminate between variability due to instructional quality and variability due to FTS brand differences.

## Conclusions

Overall, results underscore a need to address variability in FTS instructions, including variability in provision of information about FTS, specific testing methods, interpretation of test results, and additional resources. Standardized and accessible instructions, as well as continued refinement of drug checking technologies, is critical to optimizing the efficacy of FTS as a harm reduction tool and reducing accidental fentanyl exposure.

## Data Availability

Data and materials available from the corresponding author upon reasonable request.

## Abbreviations

FTS: Fentanyl Test Strip
MDMA: 3,4-Methyl​enedioxy​methamphetamine
